# Exploring the Theoretical Potential of Benzphetamine in the Treatment of Attention-Deficit/Hyperactivity Disorder (ADHD)

**DOI:** 10.7759/cureus.100363

**Published:** 2025-12-29

**Authors:** Priti M Kothari, Daniel Singer

**Affiliations:** 1 Child and Adolescent Psychiatry, Private Practice, Boca Raton, USA; 2 Harriet L. Wilkes Honors College, Florida Atlantic University, Jupiter, USA

**Keywords:** adhd pharmacotherapy, attention-deficit/hyperactivity disorder (adhd), benzphetamine, central nervous system stimulant, dextroamphetamine, methamphetamine, novel pharmacotherapy

## Abstract

Benzphetamine HCl (Didrex) is a Schedule III amphetamine indicated for the treatment of obesity. Amphetamine and methylphenidate are CNS stimulants regarded as the most effective treatment for attention-deficit/hyperactivity disorder (ADHD). Currently, both classes comprise the nationwide shortage of stimulants used to treat ADHD. Benzphetamine is a prodrug yielding dextroamphetamine (Zenzedi) and dextromethamphetamine (formerly Desoxyn). Both metabolites hold FDA approval to treat ADHD, suggesting their potential to aid in the shortage. Nonetheless, the lack of data on the compound limits its hypothetical applicability, thus prompting a theoretical review. Prior studies found dextroamphetamine to be more potent than benzphetamine and suggested the behavior of the compounds to differ; despite this, the absence of data in ADHD subjects and the limited data on obese subjects significantly limited our investigation. While benzphetamine appears mechanistically promising, the paucity of literature prevented us from drawing a theoretical claim. This editorial examined the theoretical pharmacological rationale for benzphetamine based on known mechanisms; it does not present new clinical data, efficacy claims, or treatment recommendations.

## Editorial

Benzphetamine HCl (Didrex) is a Schedule III amphetamine central nervous system (CNS) stimulant approved by the FDA in 1960. It is indicated for the treatment of short-term exogenous obesity and used as an adjunct or a monotherapy in patients 12 years and older for a maximum of 12 weeks (Pharmacia & Upjohn Company, 2009) [1]. CNS stimulants are widely considered the "gold standard" for attention-deficit hyperactivity disorder (ADHD); this results from decades of research indicating significant symptom and quality of life improvement. The two classes of CNS stimulants used in ADHD are methylphenidate and amphetamine; since 2022, a substantial production drop for both compounds has led to a continuous shortage, leaving patients with the option of attempting to find a pharmacy with an active stock or being without their medication. The debilitating shortage warrants consideration of overlooked compounds such as benzphetamine. This article examines benzphetamine on a theoretical basis and examines whether benzphetamine's pharmacology could justify future research for ADHD treatment; it does not present new clinical data, efficacy claims, or treatment recommendations. 

Benzphetamine is an active prodrug absorbed rapidly in the gastrointestinal tract before undergoing N-demethylation via cytochrome P450 enzymes, yielding metabolites dextroamphetamine (Zenzedi) and dextromethamphetamine (formerly Desoxyn). Both metabolites are FDA-indicated pharmacotherapy options for ADHD, further justifying benzphetamine's investigation (National Center for Biotechnology Information [NCBI], 2025) [2]. Similarly, lisdexamfetamine (Vyvanse) is a prodrug hydrolyzed into dextroamphetamine. Despite similarities, benzphetamine retains a proposed clinical duration of four to six hours, whereas lisdexamfetamine has a duration of 10-12 hours (NCBI, 2025) [3]; therefore, benzphetamine may be classified as an immediate-release (IR) stimulant due to its duration, which is the same as that of its metabolites. The current dosing for benzphetamine consists of 25 or 50mg tablets taken one to three times daily, with dosing not usually exceeding 150mg daily (Pharmacia & Upjohn Company, 2009) [1].

Benzphetamine is the only FDA-approved amphetamine classified as a Schedule III controlled substance, claiming a low to moderate potential of abuse; stimulants indicated for ADHD are classified as Schedule II, claiming a high potential of abuse. Schedule II controlled substances are significantly more regulated than Schedule III-IV substances; therefore, the unique scheduling could lead to more patient access.

In a human trial comparing anorexic symptoms of benzphetamine and dextroamphetamine, researchers found 40-80mg of benzphetamine to be equivalent to 5-10mg of dextroamphetamine (Veldkamp et al., 1964) [4]; Another 2017 trial found dextroamphetamine to be 180-fold more potent than benzphetamine in rhesus monkeys (Banks et al., 2017) [5]. Both trials discovered dextroamphetamine to be more potent than benzphetamine, which can be backed pharmacologically; however the significance of such cannot be drawn based on limited data.

Stimulants for the treatment of ADHD are generally approved for ages six and older. Benzphetamine has been proven safe for ages 12 and older for obesity. However, there is no data on its safety profile in ADHD subjects, posing a significant limitation. 

A randomized controlled trial (RCT) comparing single dose benzphetamine 75mg, hourly doses of 15mg over five hours (sustained release regimen), and placebo found subjects randomized to the controlled-release formulation produced smoother and more gradual responses than those randomized to 75mg in a single dose; even stating that a proper sustained release formulation may have potential (Schlagel and Sanborn, 1969) [6]. The RCT also found that benzphetamine induced an increase in total or nonfiltered EEG mean energy content; this is contrary to the findings for dextroamphetamine 15mg reported by Goldstein et al. [7] which induced a decrease. There is no explanation for this contrast; however, benzphetamine behaved consistently differently from dextroamphetamine on an EEG, suggesting the neurochemical response of the molecule to contrast with that of dextroamphetamine. This finding further prompts investigation of clinical similarities and contrasts of the compounds. However, the trial did not report adverse events or other required data regarding participants, possibly due to its occurrence in 1969, but still raises significant concern. Distinctly, this trial examined the pharmacokinetics in healthy controls as opposed to obese patients.

Benzphetamine is an overlooked amphetamine behaving as an IR medication that could be investigated amid the shortage. However, significant limitations prevent formation of a claim due to an absence of clinical data in ADHD subjects, as there is very little data produced after 1970. Most concerning, there is limited data for its approved indication of obesity. We cannot determine the drug's potential with almost exclusively pharmacologic data, despite amphetamines remaining an adequate treatment option for ADHD. Previous studies conclude that dextroamphetamine is more potent than benzphetamine and suggest contrasting behaviors of the molecules, but again, the significance is unknown. Before benzphetamine can be examined, further research focusing on the safety profile, dosing, practicality, and clinical potential must be completed.
